# Glypican-1: a modulator of neurodegenerative and tumorigenic pathways

**DOI:** 10.3389/fnins.2026.1850145

**Published:** 2026-05-15

**Authors:** Katrin Mani

**Affiliations:** Department of Experimental Medical Science, Biomedical Center A13, Lund University, Lund, Sweden

**Keywords:** Alzheimer's disease, ascorbate, glypican-1, heparan sulfate, neurodegeneration, nitric oxide, Parkinson's disease, proteostasis

## 1 Introduction

Proteoglycans are active coordinators of cellular information, dynamically integrating signals from the cell surface, extracellular matrix, and intracellular compartments. These structurally diverse glycoconjugates form a flexible layer of biological information that governs molecular recognition and tissue organization. By translating enzymatic remodeling of glycan structures into context-dependent outcomes, proteoglycans orchestrate cellular processes and maintain the balance between development, tissue homeostasis, and disease.

Within this family, the heparan sulfate (HS) proteoglycan glypican-1 (GPC1) has emerged as a pivotal and context-dependent regulator at the intersection of cancer and neurodegenerative disease. GPC1 is broadly expressed, with particularly high levels in the central nervous system and epithelial tissues, where it modulates signaling pathways governing cell communication and homeostasis (Jen et al., 2009; Hassan et al., 2021). In the brain, GPC1 localizes predominantly to neurons and astrocytes, where it regulates synaptic plasticity, proteostasis, and protein aggregation dynamics, thereby linking it to neurodegenerative pathology (The Human Protein Atlas, 2026; Oikari et al., 2020; Kamimura and Maeda, 2021). In peripheral tissues, GPC1 influences epithelial proliferation, tissue architecture, and protein homeostasis, underscoring its roles in tumor initiation and progression (Wang et al., 2019; Li et al., 2020). These diverse, context-specific functions highlight GPC1 as a potential regulatory hub through which glycan remodeling can shape disease-relevant pathways.

In this Opinion, emerging mechanistic insights into GPC1-mediated remodeling are presented, along with convergent themes and distinct, disease-specific pathways in tumorigenesis and neurodegeneration. The potential of GPC1 as a diagnostic biomarker and therapeutic target is further evaluated, emphasizing how focused study of this proteoglycan provides a framework for translating mechanistic insights into clinical applications.

## 2 Glypican-1-HS homeostasis as a regulatory node in neurodegenerative disease

GPC1 functions as a mechanistic regulator of neuronal proteostasis through dynamic remodeling of its HS chains. By interacting with misfolded proteins, influencing pathological phosphorylation, and coordinating intracellular trafficking pathways, GPC1 links glycan metabolism to neurodegenerative processes (Watanabe et al., 2004; Reinhard et al., 2013; Ozsan McMillan et al., 2023; Cheng et al., 2024).

In Alzheimer's disease (AD), experimental studies show that cell-surface GPC1 undergoes co-internalization with amyloid precursor protein (APP) and influences APP processing. Within endosomal compartments, GPC1 releases HS oligosaccharides that modulate proteolytic APP cleavage. Specifically, β-secretase-mediated processing of APP generates the β-N-terminal fragment (β-NTF), which stimulates copper-, nitric oxide-, and ascorbate-dependent (Cu/NO-ascorbate-dependent) HS release from GPC1. The released HS oligosaccharides subsequently inhibit β-secretase activity and promote the trafficking of amyloid-β together with HS toward nuclear, autophagosomal, and lysosomal degradation pathways (Cheng et al., 2020, 2021).

GPC1-mediated HS dynamics influence key pathogenic processes in AD across genetic, environmental, and inflammatory contexts. In neuronal cells, the ApoE4 genotype or exposure to the cyanobacterial neurotoxin β-*N*-methylamino-l-alanine (BMAA) disrupts GPC1 HS processing, enhancing APP proteolytic cleavage and promoting the accumulation of toxic degradation products (Cheng et al., 2019, 2021). Similarly, proinflammatory cytokines drive the accumulation of GPC1-derived HS and β-cleaved APP fragments in autophagosomes, linking inflammatory stress to impaired neuronal proteostasis (Cheng et al., 2020). Notably, these pathogenic effects can be mitigated by ascorbate, which induces HS release from GPC1 and promotes the clearance of APP degradation products, including those associated with ApoE4-dependent stress (Cheng et al., 2021). Collectively, these findings establish GPC1-HS remodeling as a central pathway that integrates genetic, environmental, and inflammatory factors to regulate proteostasis and amyloidogenic processing in neurons.

Importantly, the regulatory effects of GPC1-derived HS extend beyond amyloid-β metabolism. Activation of the GPC1-HS pathway attenuates α-synuclein oligomerization and reduces tau hyperphosphorylation (pTau181 and pTau217) in experimental models of Parkinson's and Alzheimer's disease (Cheng et al., 2022, 2023b, 2024, 2025). Consistent with this, silencing GPC1 expression increases amyloidogenic protein accumulation, whereas pharmacological stimulation—via ascorbate, copper, or nitric oxide donors—reduces the formation of toxic oligomeric species (Cheng et al., 2023b, 2024, 2025).

Together, these findings position the GPC1–HS axis as a convergent regulator of protein aggregation and clearance pathways in neurodegenerative disease. By linking GPC1 remodeling to APP processing and broader proteostatic mechanisms, GPC1 integrates multiple pathogenic processes, underscoring its potential as a mechanistically grounded therapeutic target.

## 3 Glypican-1 in cancer: a central modulator of tumor growth

GPC1 has emerged as a key regulator of tumor initiation, progression, and metastasis, bridging intracellular proteostasis and extracellular oncogenic signaling (Wang et al., 2019). Similar to neuronal cells, cancer cells accumulate oxidized proteins (Selvaraj et al., 2025), and GPC1 participates in their clearance (Mani et al., 2007; Fransson and Mani, 2007). Cell-surface GPC1 is internalized and transported to endosomes, where its HS chains are cleaved through a Cu/NO-ascorbate-dependent reaction, releasing free polyanionic HS oligosaccharides. These HS oligosaccharides form conjugates with oxidized proteins via Amadori rearrangement and target them to cellular degradation pathways. The GPC1 core protein is then glycosylated in the Golgi, redecorated with HS chains, and recycled to the cell surface, completing a dynamic proteostasis cycle (Mani et al., 2007).

Beyond its role in maintaining cellular proteostasis, GPC1 organizes extracellular growth factors and morphogens to modulate oncogenic signaling, including Wnt, FGF, Hedgehog, and TGF-β pathways (Hassan et al., 2021). Through this scaffolding function, GPC1 regulates cellular proliferation, migration, and epithelial–mesenchymal transition (EMT) (Kaur and Cummings, 2019; Li et al., 2020).

Increasing evidence links aberrant GPC1 expression to multiple malignancies, including pancreatic, esophagus, prostate, breast, and bladder cancers, as well as glioma and hepatocellular carcinoma, where it exerts pleiotropic effects and has emerged as a potential diagnostic and prognostic biomarker (Melo et al., 2015; Qian et al., 2018; Zhao et al., 2022; Harada et al., 2017; Levin et al., 2018; Grillo et al., 2021; Chen et al., 2020; Cheng et al., 2023a).

Mechanistic studies show that GPC1 modulates receptor–ligand interactions and downstream kinase activation across multiple oncogenic pathways, including FGF, VEGF, HGF, TGF-β, Wnt, BMP, and Hedgehog (Lund et al., 2020; Pan and Ho, 2021; Cheng et al., 2023a). In addition, GPC1 facilitates extracellular vesicle mediated transfer of signaling proteins, promoting the intercellular transfer of oncogenic signals and contributing to metastatic niche formation (Christianson et al., 2013; Melo et al., 2015; Zhao et al., 2022). Functional perturbation of GPC1—through genetic silencing, overexpression, or antibody-mediated inhibition—alters tumor cell proliferation, migration, and invasion (Harada et al., 2017; Cheng et al., 2023a).

Clinically, circulating GPC1 has emerged as a promising biomarker in pancreatic and prostate cancers, and ongoing clinical studies are evaluating its association with clinicopathological parameters in pleural mesothelioma and lung cancer (Zhou et al., 2018; Levin et al., 2018; ClinicalTrials.gov, 2026). Detection of GPC1 in blood can be achieved through exosome isolation or enzyme-linked immunosorbent assays, while immunohistochemical analyses of tumor biopsies may provide complementary prognostic information and identify potential tumor antigens (Frampton et al., 2018; Zhou et al., 2018; Levin et al., 2018). These findings underscore the translational potential of targeting GPC1, particularly in the context of precision oncology, and support systematic exploration of GPC1 as both a mechanistic driver of tumor biology and a clinically actionable biomarker.

## 4 Shared mechanistic themes across neurodegeneration and cancer

Despite their divergent clinical manifestations, cancer and neurodegenerative diseases share underlying regulatory principles, with GPC1 emerging as a unifying molecular node. In both contexts, GPC1 activity intersects with oxidative stress, inflammatory pathways, and signal transduction networks—core modulators of cellular homeostasis and disease progression. Neurons and cancer cells alike accumulate oxidized and misfolded proteins under metabolic and oxidative stress, placing substantial demands on proteostasis mechanisms (Selvaraj et al., 2025).

In neurons, GPC1-mediated Cu/NO-ascorbate-dependent HS release regulates amyloid-β formation and clearance, tau hyperphosphorylation, and α-synuclein aggregation, maintaining neuronal proteostasis (Cheng et al., 2020, 2022, 2024). This pathway is sensitive to multiple stressors: the ApoE4 genotype, exposure to neurotoxins such as β-*N*-methylamino-l-alanine, and proinflammatory cytokines all disrupt HS dynamics, enhancing APP processing and accumulation of toxic protein species (Cheng et al., 2019, 2020, 2021). Importantly, ascorbate-induced HS release can counteract these effects, highlighting the integrative role of GPC1-HS remodeling in linking genetic, environmental, and inflammatory stress to neuronal proteostasis.

In cancer cells, similar redox-sensitive mechanisms facilitate the clearance of oxidized and misfolded proteins, with GPC1 directing HS-associated cargo to proteasomal and autophagic degradation pathways. Beyond proteostasis, GPC1 modulates extracellular signaling and cellular responses to environmental inputs. In tumors, HS chains on GPC1 scaffold growth factor gradients, regulate receptor signaling, and facilitate extracellular vesicle-mediated communication, influencing proliferation, migration, and EMT in a context-dependent manner. Redox-sensitive HS modifications further integrate metabolic and inflammatory signals with extracellular matrix dynamics, positioning GPC1 as a contextual molecular switch that translates microenvironmental cues into disease-specific responses.

This convergence illustrates a broader principle: GPC1 functions as a central integrator of proteostasis, signaling, and microenvironmental sensing across organ systems. Its context-dependent HS remodeling allows a single proteoglycan to coordinate responses to genetic, environmental, and inflammatory stress across neurodegenerative and tumor contexts. Recognizing these shared mechanisms opens opportunities for cross-disciplinary therapeutic strategies targeting GPC1-HS dynamics, with potential implications for both neurodegenerative disease and cancer.

However, this apparent convergence also reveals a fundamental therapeutic paradox. While activation of GPC1-HS dynamics promotes proteostatic clearance and appears beneficial in neurodegenerative settings, its effects in cancer are more complex, as GPC1 simultaneously supports tumor growth, signaling, and metastatic progression. Thus, modulation of GPC1 may exert potentially opposing effects across disease settings. Importantly, these shared mechanisms do not imply uniform therapeutic strategies, as GPC1 can be targeted through distinct and disease-specific intervention modalities.

## 5 Concluding perspectives and future directions

GPC1 emerges as a mechanistically versatile proteoglycan at the intersection of neurodegeneration and cancer, integrating intracellular proteostasis, extracellular signaling, and environmental sensing. Its dual role highlights the potential to translate mechanistic insights into targeted therapeutic strategies. In neurodegenerative disease, interventions that modulate GPC1-HS dynamics—such as ascorbate/vitamin C or other redox-active agents—may enhance clearance of amyloidogenic proteins and attenuate amyloid-β, tau and α-synuclein pathology, particularly in individuals carrying the ApoE4 risk allele. Tailoring such interventions according to genetic background and environmental exposure could provide a precision-medicine framework for mitigating sporadic and late-onset Alzheimer's disease.

In cancer, GPC1 represents both a prognostic factor and a therapeutic target. Its roles in scaffolding growth factor gradients and regulating receptor signaling support its potential as a biomarker for tumor progression, metastasis, and treatment response. Antibody-mediated inhibition, genetic silencing, or small-molecule modulators of GPC1-HS dynamics may complement existing therapies by targeting both intracellular proteostasis and extracellular oncogenic signaling.

A critical challenge emerging from these observations is the context-dependent duality of GPC1 function. Mechanistic evidence suggests that enhancing GPC1-HS dynamics may be beneficial in neurodegenerative disease by promoting the clearance of amyloidogenic proteins. In contrast, in cancer, GPC1 supports tumor cell proliferation, migration, and oncogenic signaling. This raises a fundamental therapeutic paradox: strategies aimed at enhancing GPC1 activity in neurodegeneration may, in principle, promote tumor-supportive processes, whereas inhibition of GPC1 in cancer could interfere with neuronal proteostatic functions. Importantly, these opposing effects may be reconciled by fundamentally distinct therapeutic strategies in each disease context. In cancer, GPC1 is primarily explored as a prognostic biomarker and as a target for antibody-mediated inhibition or other strategies that block extracellular signaling functions. In contrast, in neurodegenerative disease, therapeutic approaches focus on stimulating GPC1-dependent HS release—through redox-active compounds such as ascorbate or nitric oxide donors—to enhance intracellular proteostatic clearance. These mechanistically distinct intervention strategies support disease-specific modulation of GPC1, potentially minimizing adverse cross-disease effects.

Future research should aim to systematically integrate GPC1 status, HS remodeling capacity, and individual genetic risk factors into disease stratification. Such an approach could guide the design of cross-disease interventions, linking neurodegenerative and oncologic therapeutics, and foster the development of mechanistically informed biomarkers and precision-targeted therapies. Ultimately, leveraging GPC1 as both a diagnostic and therapeutic node offers a promising avenue for interventions that address the convergent mechanisms underlying seemingly disparate human diseases.
